# Robotic versus laparoscopic major hepatectomy for hepatocellular carcinoma: short-term outcomes from a single institution

**DOI:** 10.1186/s12893-022-01882-8

**Published:** 2022-12-17

**Authors:** Linsen Liu, Yan Wang, Tianchong Wu, Jianwei Lin, Lingna Deng, Jiling Jiang, Tailai An

**Affiliations:** 1 Department of Hepatobiliary and Pancreatic Surgery, Shenzhen People’s Hospital, Dongmen North Road 1017, Luohu District, Shenzhen, 518020 Guangdong People’s Republic of China; 2 Department of Radiology, Shenzhen People’s Hospital, Dongmen North Road 1017, Luohu District, Shenzhen, 518020 Guangdong People’s Republic of China; 3 Department of Pathology, Qing Yuan People’s Hospital, Yinquan Road B24, Qingcheng District, Qingyuan, 511518 Guangdong People’s Republic of China; 4 The First Department of Surgery, Shenzhen Traditional Chinese Medicine Hospital, Fuhua Road 1, Futian District, Shenzhen, 518033 Guangdong People’s Republic of China

**Keywords:** Robotic major hepatectomy, Laparoscopic major hepatectomy, Intraoperative and postoperative results

## Abstract

**Background:**

Currently, an increasing number of robotic major hepatectomies for hepatocellular carcinoma (HCC) are being performed. Despite the advantages of robotic surgery over laparoscopic procedures, studies comparing robotic with laparoscopic major hepatectomy in terms of short-term results remain scarce. This study was performed to compare robotic major hepatectomy and laparoscopic major hepatectomy in terms of their intraoperative and postoperative results.

**Methods:**

Data regarding demographics and intraoperative and postoperative results of 131 patients undergoing robotic or laparoscopic major hepatectomy between January 2017 and March 2022 were retrieved from their medical records and compared between the two types of surgery.

**Results:**

Between January 2017 and March 2022, 44 robotic major hepatectomies and 87 laparoscopic major hepatectomies were performed at the Department of Hepatobiliary and Pancreatic Surgery, Shenzhen People’s Hospital. Patients undergoing robotic major hepatectomy were not significantly different from those undergoing laparoscopic major hepatectomy in terms of age (P = 0.397), sex (P = 0.624), body mass index (BMI) (P = 0.118), alpha-fetoprotein (AFP) (P = 0.09), tumor size (P = 0.176), cirrhosis (P = 0.384), fatty liver (P = 0.162), preoperative antiviral treatment (P = 0.934), hepatitis B virus (HBV) DNA (P = 0.646) and operation type (P = 0.054). Robotic major hepatectomy was associated with a longer operation time (median: 255.5 versus 206.8 min; P < 0.001) and less estimated blood loss (median: 118.9 versus 197.0 ml; P = 0.002) than laparoscopic major hepatectomy. However, robotic major hepatectomy was not significantly different from laparoscopic major hepatectomy regarding length of postoperative hospital stay (P = 0.849), open conversion (P = 0.077), ICU stay (P = 0.866), postoperative massive abdominal bleeding (P = 1.00), portal vein thrombosis (P = 1.00), abdominal infection (P = 1.00), pulmonary infection (P = 1.00), pulmonary embolism (P = 1.00), cardiac complications (P = 1.00), liver failure (P = 1.00), kidney failure (P = 1.00), biliary leak (P = 1.00), positive resection margin (P = 1.00), 30-day mortality (P = 1.00) and 90-day mortality (P = 1.00).

**Conclusions:**

Robotic major hepatectomy was as effective as laparoscopic surgery in terms of intraoperative and postoperative results but took longer and could more efficiently control intraoperative blood loss.

## Background

Laparoscopy techniques have been widely applied in the resection of malignant and benign tumors of the liver [1–5]. In clinical practice, three types of laparoscopic hepatectomy, including pure laparoscopic, hand-assisted laparoscopic, and hybrid approaches, have been developed [6, 7]. Previously, due to less advanced nature of the surgical techniques, early laparoscopic resections were usually nonanatomic wedge resections of peripheral lesions, while some more recent studies have reported that anatomic major hepatectomy could be safely accomplished by experienced surgeons [8–10]. In some previously published studies, it was reported that in comparison with open surgery, laparoscopic hepatectomy was associated with significantly less blood loss, less severe postoperative pain, a shorter length of hospital stay, improved cosmesis and greater cost-effectiveness [11–15]. Additionally, some other studies have also demonstrated that for patients with hepatocellular carcinoma (HCC) or metastatic colorectal cancer (CRC), laparoscopic hepatectomy resulted in a comparable R0 resection rate and 5-year overall survival [16–18].

However, for complex liver surgery, widespread application of laparoscopic surgery remains a significant challenge [19–21]. A few limitations of laparoscopic hepatectomy have been described. First, unlike in open surgery, the movement of laparoscopic instruments is significantly restricted (4 degrees of freedom in laparoscopic surgery versus 7 degrees of freedom of the human wrist in open surgery) [22]. Second, a satisfactory three-dimensional perspective can not be provided by laparoscopy [22]. Third, during laparoscopic surgery, hand tremor is enhanced due to the length of the instruments, and imperfect ergonomics is commonly encountered [22]. In one study by Nguyen et al., 65% of all laparoscopic hepatic resections were left sectionectomies or nonanatomic resections, whereas only 9%, 7%, and 1% were major anatomic right, left, and extended hepatectomies, respectively [2], indicating potential difficulties encountered during laparoscopic hepatectomies. However, these limitaitons have been remarkably improved due to the significant improvements in the skills of surgeons, and an increasing number of major laparoscopic hepatectomies are being performed each year [23].

Initially introduced in the late 1980s for military purposes, robotic surgery has become a focus of numerous research and development efforts. Theoretically, the advantages of robotic surgery include improved dexterity and precision, higher visual magnification and markedly decreased tremor and fatigue [22, 24]. Early experiments performed in a porcine model included cholecystectomy, choledochotomy, T-tube placement and repair of the bile duct [25]. The first reported robotic hepatic surgery performed on a patient was accomplished by surgeons from the Czech Republic [26]. Over the last decade, case reports, single-center series and large-scale studies have been published on robotic hepatic surgery [27–30]. Despite the encouraging results reported by these studies, studies comparing robotic hepatectomy and laparoscopic hepatectomy in terms of long-term and short-term outcomes are still scarce. Furthermore, in most studies comparing robotic hepatectomy and laparoscopic hepatectomy, both minor hepatectomy and major hepatectomy were included together. Therefore, studies comparing robotic major hepatectomy and laparoscopic major hepatectomy are still needed to help surgeons choose how hepatectomy should be performed.

This study was performed to summarize our experience with robotic major hepatectomy for HCC at the Department of Hepatobiliary and Pancreatic Surgery, Shenzhen People’s Hospital, since it was first applied in 2020 and to compare the results with those of established laparoscopic hepatectomy. Additionally, we also reviewed the most recent studies on robotic hepatectomy.

## Materials and methods

### Patients

The present study was a retrospective study assessing the short-term outcomes of patients with HCC who underwent robotic or laparoscopic major hepatectomy at the Department of Hepatobiliary and Pancreatic Surgery, Shenzhen People’s Hospital between January 2017 and March 2022. During this period, a total of 87 patients underwent laparoscopic major hepatectomy, and 44 underwent robotic major hepatectomy. The following clinicopathological variables were retrieved from the medical record system: age, sex, body mass index (BMI), alpha-fetoprotein (AFP), tumor size, cirrhosis, fatty liver, preoperative antiviral treatment, hepatitis B virus (HBV) DNA, operation type, estimated blood loss (EBL), operation time, length of hospital stay after hepatectomy, open conversion, intensive care unit (ICU) stay, postoperative complications, 30-day mortality, 90-day mortality and resection margin status. Both laparoscopic major hepatectomy and robotic major hepatectomy were accomplished as previously described [2, 31]. Major hepatectomy was defined as resection of 3 or more liver segments. Treatment strategies were made at a case discussion conference, and a multidisciplinary teamwork framework was adopted. Lesion location, overall performance status, liver function preservation and future liver remnant (FLR)/standard liver volume (SLV) were the main factors determining the range of resection. Conclusions drawn by the case discussion conference were final. For each patient scheduled to undergo major hepatectomy, the robotic or laparoscopic approach was chosen by the patient after the surgeon explained the cost, advantages and disadvantages of robotic and laparoscopic hepatectomy in detail. Major conditions specifically contraindicating robotic or laparoscopic major hepatectomy include a history of upper abdominal surgery or perforating diseases that may significantly increase the risk of extensive adhesion. Moreover, the surgeries in this study were performed by surgeons undergoing the same training programs.

Data regarding history, demographics, and intraoperative and postoperative results were obtained from the patients’ medical records. Intraoperative results such as EBL were identified from anesthesia and operative records. Operation time (OT) was defined as the duration between skin incision and wound closure. Postoperative ICU stay was confirmed by browsing the discharge summary. Discharge summaries and cover sheets were also browsed to confirm postoperative complications, and postoperative complications were graded using the Clavien‒Dindo classification system [32]. Major complications were defined as events that should be treated with surgery or endoscopic or radiological interventions (Clavien‒Dindo classification grade ≥ 3).

### Definition of massive abdominal bleeding and liver failure

As massive abdominal bleeding and liver failure are the two most sever complications after hepatectomy, we specified their definitions for this study. Posthepatectomy liver failure was defined as the impaired ability of the liver to maintain its synthetic, excretory, and detoxifying functions, as characterized by an increased international normalized ratio and concomitant hyperbilirubinemia (according to the normal limits of the local laboratory) on or after postoperative day 5. The following criteria were used to define posthepatectomy liver failure: persistently elevated level of bilirubin > 100 mmol/L (i.e., 5.8 mg/dL), prothrombin time > 24 s [or requiring daily fresh frozen plasma (FFP)], and elevated levels of aminotransferase with associated encephalopathy. The following criteria were adopted to diagnose massive abdominal bleeding: more than 100 ml blood drained out per hour, signs of shock, and more than 1000 ml transfused within 4 h.

### Surgical procedures

Laparoscopic hepatectomy is a mature technique, the surgical procedures of which were consistent with some previous studies [33, 34]. Similar to laparoscopic major hepatectomy, all inflow-occlusion techniques adopted in open major hepatectomy could be utilized during robotic major hepatectomy. The most commonly adopted method was the Pringle method. The rubber band plus Hemolok device was commonly used. Using the rubber band plus Hemolok, we repeatedly occluded the first porta hepatis. For robotic major hepatectomy, we usually adopted the “first porta hepatis” method. The hepatic artery and portal vein supplying the lobes where HCC lesions were located were carefully exposed and dissected to minimize blood loss during parenchyma division. The low central vein pressure technique has been widely accepted at most centers to minimize blood loss during hepatectomy. However, at our center, the low central vein pressure technique is not routinely used in all patients. Low central vein pressure is adopted when it was expected that a large hepatic vein would be managed or tumors located in the caudate lobe or caudate process were to be resected. At our center, during surgery for these patients, a blocking band was placed in case of emergent situations. Conventionally, a three-arm robotic system were used to perform major hepatectomy. Even when the hemorrhage was not easy to address, the third arm was used to compress sites of bleeding, while the other two arms were used to perform sutures or other treatments.

To date, an increasing number of devices have been developed for dividing the parenchyma. Regardless of the device used, the purpose of properly treating vessels and bile ducts remains unchanged. Unfortunately, during robotic liver surgery, the parenchyma division devices we could choose were limited, and the main tools we used were ultrasonic scalpels and Maryland bipolar electrocoagulation forceps. The ultrasonic scalpel was mainly used to disconnect the liver plane in a straight line. Since the ultrasonic scalpel could not be freely bent like other instruments, in certain situations, Maryland bipolar electrocoagulation forceps were to divide the hepatic parenchyma in a nonstraight plane or to manage parts that the ultrasonic scalpel could not reach. For a hepatic parenchyma within 1 cm beneath the surface, the slow speed gear of the ultrasonic scalpel could be used to perform dissection since at these depths, there are no large pipeline structures. However, for deep hepatic parenchymas or those located in the visceral surface, dissection was performed using the fast speed gear of the ultrasonic scalpel (shallow to deep and inferior to superior), and some hepatic parenchyma was smashed due to vibration of the ultrasonic scalpel, while the rest was smashed by closed forceps. For pipeline structures encountered during parenchyma division, due to magnification effects, it was not easy to accurately assess the diameter. Pipelines smaller than the coagulating shear were directly managed by the scalpel in the slow speed gear, while those larger than the coagulating shear (especially those larger than 3 mm) were carefully ligated using titanium or Hemolok clips. For extremely thick vessels, such as the right hepatic vein, left hepatic vein or middle hepatic vein, linear cutting closure devices were used. However, sometimes, to reduce costs, we did not use linear cutting closure devices, and instead occluded the large vessels using Hemolok clips after they were constricted by silk sutures. It should be emphasized that we did not advocate direct clamping of these extremely large vessels using Hemolok clips because direct clamping might lead to uncontrollably massive bleeding. Sometimes, accidents might occur during parenchyma division. If vessels in the liver section retracted after dissection or small tears formed on the surface of large vessels due to blunt separation, it was impossible to achieve ideal hemostasis by clamping or electric coagulation. Under these circumstances, the superiority of robotic surgery seemed more apparent, given its increased dexterity and three-dimensional visibility, tremor limiting and comfortable operating platform. These advantages of robotic surgery could overcome the inherent shortcomings of laparoscopic surgery, and the surgeons could perform stable and accurate suturing in a narrow space. During the whole process of parenchyma division, hemorrhage was effectively managed to maintain a clean operative field, since bleeding could not only affect the surgeons’ mood but also lead to a less satisfying and safe division process.

### Ethical approval

This study was approved by the Ethical Committee, Shenzhen People’s Hospital. All patients provided written informed consent. The Declaration of Helsinki was adhered to during the whole process of this study [35].

### Statistical analysis

Statistical Package for the Social Sciences 22.0 (SPSS 22.0, SPSS Inc, Chicago, IL) was used to perform the statistical analyses involved in this study. Categorical variables are presented as numbers and percentages, while continuous variables are presented as the means with standard deviations. Categorical variables were analyzed using the chi^2^ test, while continuous variables were analyzed by the t test. All the statistical analyses performed in this study were two-sided in nature, and P values < 0.05 were considered statistically significant.

## Results

### Baseline characteristics

Patients undergoing robotic major liver resection (n = 44) were compared with those undergoing laparoscopic major liver resection (n = 87) in terms of baseline characteristics. The results demonstrated that the two groups were not significantly different regarding age (P = 0.397), sex (P = 0.624), BMI (P = 0.118), AFP (P = 0.09), tumor size (P = 0.176), cirrhosis (P = 0.384), fatty liver (P = 0.162), preoperative antiviral treatment (P = 0.934), HBV DNA (P = 0.646) and operation type (P = 0.054) (Table 1).

**Table 1:** Comparisons between robotic major hepatectomy and laparoscopic major hepatectomy regarding baseline characteristics

Characteristics	No	Laparoscopic	Robotic	χ^2^/t	P value
		(n = 87)	(n = 44)		
Age		51.49 ± 13.27	49.82 ± 9.06	0.85	0.397
Sex					
Male	90	61	29	0.24	0.624
Female	41	26	15		
BMI		22.28 ± 3.61	23.29 ± 3.12	− 1.575	0.118
AFP					
< 20	95	59	36	2.875	0.090
> 20	36	28	8		
Tumor size					
< 5 cm	82	58	24	1.834	0.176
≥ 5 cm	49	29	20		
Cirrhosis					
No	44	27	17	0.757	0.384
Yes	87	60	27		
Fatty liver					
No	111	71	40	1.954	0.162
Yes	20	16	4		
Preoperative antiviral treatment					
No	84	56	28	0.007	0.934
Yes	47	31	16		
HBV DNA					
≤ 10^4^	81	55	26	0.211	0.646
> 10^4^	50	32	18		
Operation type					
Right hemihepatectomy	51	40	11	9.303	0.054
Left hemihepatectomy	53	28	25		
Central bisectionectomy	18	14	4		
Extended right hemihepatectomy	5	3	2		
Extended left hemihepatectomy	4	2	2		

^*^P < 0.05: statistical significance

### Robotic major hepatectomy versus laparoscopic major hepatectomy in terms of short-term outcomes

For patients undergoing robotic or laparoscopic major hepatectomy, surgery-related, postoperative and oncological results are presented in Table 2. Significant differences were observed in operation time (206.8 ± 69.2 versus 255.5 ± 56.3 min, P < 0.001) in favor of laparoscopic major hepatectomy. However, the two groups were not significantly different from each other in terms of length of postoperative hospital stay (P = 0.849), open conversion (P = 0.077), ICU stay (P = 0.866), postoperative massive abdominal bleeding (P = 1.00), portal vein thrombosis (P = 1.00), abdominal infection (P = 1.00), pulmonary infection (P = 1.00), pulmonary embolism (P = 1.00), cardiac complications (P = 1.00), liver failure (P = 1.00), kidney failure (P = 1.00), biliary leak (P = 1.00), positive resection margin (P = 1.00), 30-day mortality (P = 1.00) and 90-day mortality (P = 1.00). Additionally, we also revealed that the estimated blood loss during laparoscopic major hepatectomy was significantly greater than that during robotic major hepatectomy (197.0 ± 186.3 versus 118.9 ± 99.1, P = 0.002).

**Table 2:** Comparisons between robotic major hepatectomy and laparoscopic major hepatectomy regarding intraoperative and postoperative outcomes

Outcomes	Laparoscopic (n = 87)	Robotic (n = 44)	χ^2^/t	P value
Length of postoperative hospital stay	10.3 ± 3.5	10.2 ± 2.2	0.190	0.849
Open conversion	5	2	3.124	0.077
ICU stay	2	2	0.028	0.866
Postoperative massive abdominal bleeding I/II/IIIa/IIIb/IVa/IVb/V	2 0/0/0/1/1/0/0	1 0/0/0/0/1/0/0	0	1.00
Portal vein thrombosis	0	0	0	1.00
Abdominal infection	0	0	0	1.00
Pulmonary infection	0	0	0	1.00
Pulmonary embolism	0	0	0	1.00
Cardiac complications	0	0	0	1.00
Liver failure C-D I/II/IIIa/IIIb/IVa/IVb/V	3 0/0/0/0/3/0/0	1 0/0/0/0/1/0/0	0	1.00
Kidney failure C-D I/II/IIIa/IIIb/IVa/IVb/V	1 0/0/0/0/1/0/0	0 0/0/0/0/0/0/0	0	1.00
Biliary leak	0	0	0	1.00
30-day mortality	0	0	0	1.00
90-day mortality	0	0	0	1.00
Positive resection margin	0	0	0	1.00
Estimated blood loss	197.0 ± 186.3	118.9 ± 99.1	3.133	0.002
Operation time (min)	206.8 ± 69.2	255.5 ± 56.3	− 4.036	0.000

*P < 0.05: statistical significance; C–D: Clavien‒Dindo classification

## Discussion

Unlike colorectal, gynecological and urological surgeries, the laparoscopic approach has not been widely adopted in liver surgery, and most laparoscopic liver surgeries have been performed in large tertiary care centers. Concerns about laparoscopic hepatectomy mainly lie in the complexity of its procedures, difficulties in controlling massive bleeding, and a significantly steep learning curve [36, 37]. Additionally, according to a previously published study reviewing a large number of laparoscopic liver surgeries performed around the globe, the majority of minimally invasive hepatectomies were nonanatomic wedged resections for lesions located in peripheral segments [2]. With the maturation of laparoscopic techniques, an increasing number major laparoscopic liver surgeries are being performed each year. More studies are needed to elucidate the roles of the laparoscopic approach in liver surgery. An increasing number of studies have favored laparoscopic hepatectomy since in comparison with open surgery, the laparoscopic approach was associated with much better short-term outcomes and similar long-term outcomes [11–15]. However, some disadvantages of laparoscopic surgery have also been reported. According to a study by Rodrigues TFDC et al., the limited image amplification, two-dimensional tremor, the fulcrum effect, limited freedom of movement and low ergonomics of laparoscopic hepatectomy could be overcome by a robotic approach [38].

In the present study, we performed a matched comparison between laparoscopic major hepatectomy and robotic major hepatectomy. Robotic major hepatectomy was not significantly different from laparoscopic major hepatectomy in terms of most short-term outcomes except operation time and estimated blood loss during surgery. Of these short-term outcomes, resection margin status is the most important factor indicating the quality of oncological surgery, and in this study, the rate of positive resection margins after robotic major hepatectomy was not significantly different from that after laparoscopic major hepatectomy, meaning that oncological quality was not compromised due to technical reasons. The longer time it took to perform robotic major hepatectomy was expected, given that additional time was needed to dock the robot, to install the instruments and to redock or reposition the instruments if a change to the viewing field was needed. In a study by Allan Tsung et al., it was reported that early robotic hepatectomy took longer than laparoscopic hepatectomy, while late robotic hepatectomy was not significantly different from laparoscopic hepatectomy in terms of operation time [21]. Thus, considering these findings, we may conclude that the difference in operation time is caused by the learning curve, which can be overcome, similar to the results reported reported by other studies [39, 40].

Since bleeding control is the most difficult aspect of minimally invasive major liver surgeries, a robotic approach may significantly reduce blood loss during major hepatectomy due to the ease with which blood vessel bleeding can be prevented. Several characteristics of robotic surgery could contribute to preventing the bleeding of major vessels. First, the EndoWrist technology and the three-dimensional optics of the robotic system can effectively avoid injury to major vessels. Second, using the robotic system, surgeons can easily control extrahepatic inflow before transection, while during laparoscopic hepatectomy, we prefer use of a stapler to control the portal vein; however, the ideal angle for firing the stapler is not always easy to obtain, which may result in injury to other vessels during this process. Unlike in laparoscopic hepatectomy, the increased dexterity and three-dimensional field of view of robotic surgery help surgeons perform a much safer portal vein dissection and allow them to control major vessels using suture ligation rather than staples, avoiding the difficulties in placing the latter. Additionally, the increased movement freedom of movement could also enable surgeons to perform safer dissections posterior to the right hepatic vein or middle/left hepatic vein common trunk to achieve better control of extrahepatic venous outflow. Apart from better inflow and outflow control, the significantly improved magnification and field of view provided by robotic surgery enable surgeons to more accurately identify vessels for blood control and ligation. In fact, in this study, we demonstrated that robotic major hepatectomy was more efficient than laparoscopic major hepatectomy in controlling bleeding.

We then compared robotic major hepatectomy with laparoscopic major hepatectomy in terms of other short-term outcomes, such as length of hospital stay after the operation, open conversion, ICU stay, postoperative massive abdominal bleeding, portal vein thrombosis, abdominal infection, pulmonary infection, pulmonary embolism, cardiac complications (such as heart failure, cardiac arrhythmia), liver failure, kidney failure, biliary leak, 30-day mortality and 90-day mortality, revealing that robotic major hepatectomy was not significantly different from laparoscopic major hepatectomy in these regards. Thus, considering all these results, we may conclude that the two surgical approaches were not significantly different except for operation time and estimated blood loss and that robotic hepatectomy could better control intraoperative bleeding. However, these conclusions need to be verified with more randomized controlled studies.

Some inherent shortcomings of this study should be acknowledged. First, all the data involving demographics, clinicopathological variables, and intraoperative and postoperative outcomes were retrospectively retrieved from the patients’ medical records. Therefore, these data, recorded by different individuals from various departments, were subject to selection biases. Second, a relatively small number of patients were included in this study, and a larger study involving more patients is necessary to minimize the underestimation regarding the significance between the robotic group and laparoscopic group. Third, in this study, the surgical modality was not chosen randomly. Thus, likely differences in selecting patients and/or determining the surgical approach between the robotic group and laparoscopic group were not accounted for in this study. However, these potential selection biases have been minimized, since all the patients had to at least meet the indications for minimally invasive liver resection. Fourth, each robotic liver resection at our center was performed by senior surgeons. Thus, the results of our study are unlikely to be applicable to centers without surgeons sufficiently experienced in complex minimally invasive liver surgery since both robotic and laparoscopic major hepatectomy should be performed at centers by experienced general surgeons. Fifth, we did not assess whether the surgical approach affected the overall and recurrence-free survival of HCC patients. Further studies are needed to overcome these aforementioned shortcomings. Prospective randomized controlled multicenter studies should be performed to further compare robotic major hepatectomy and laparoscopic major hepatectomy.

Although we demonstrated that robotic hepatectomy could significantly reduce estimated blood loss, we did not reveal a significantly shorter operation time, reduced complications or shorter hospital stay. The downsides of robotic hepatectomy included added costs (at our center, an additional 30,000 Yuan is needed to accomplish robotic hepatectomy) and longer operation times. However, the results of this study suggest that through robotic approaches, surgeons could successfully complete a major hepatectomy purely minimally invasively. In conclusion, robotic major hepatectomy was demonstrated to have comparable feasibility and safety to laparoscopic major hepatectomy, but our findings should be further verified by more in-depth studies.

## Conclusion

In conclusion, robotic major hepatectomy was demonstrated to have comparable feasibility and safety to laparoscopic major hepatectomy, but our findings should be further verified by more in-depth studies.

## Data Availability

The datasets used and/or analyzed during the current study are available from the corresponding author on reasonable request.

## Abbreviations

HCC: Hepatocellular carcinoma
ASA: American Society of Anesthesiologists
BMI: Body mass index
AFP: Alpha-fetoprotein
ICU: Intensive care unit
ICGR15: Indocyanine green rate 15
CRC: Colorectal cancer
EBL: Estimated blood loss
OT: Operative time
SPSS22: Statistical Product and Service Solutions 22
IQR: Interquartile range
FLR: Future liver remnant
SLV: Standard liver volume
